# Supplementary material for: The causal role of circulating inflammatory markers in osteoporosis: a bidirectional Mendelian randomized study
Source: Front Immunol. 2024 Jul 18;15:1412298. doi: 10.3389/fimmu.2024.1412298 (PMC11291241; doi:10.3389/fimmu.2024.1412298)
Supplement: Supplementary file 1 [file DataSheet_1.zip › supplementary material/OSTEOPOROSIS-CIM/GCST90274825.tsv.gz_forest.pdf]

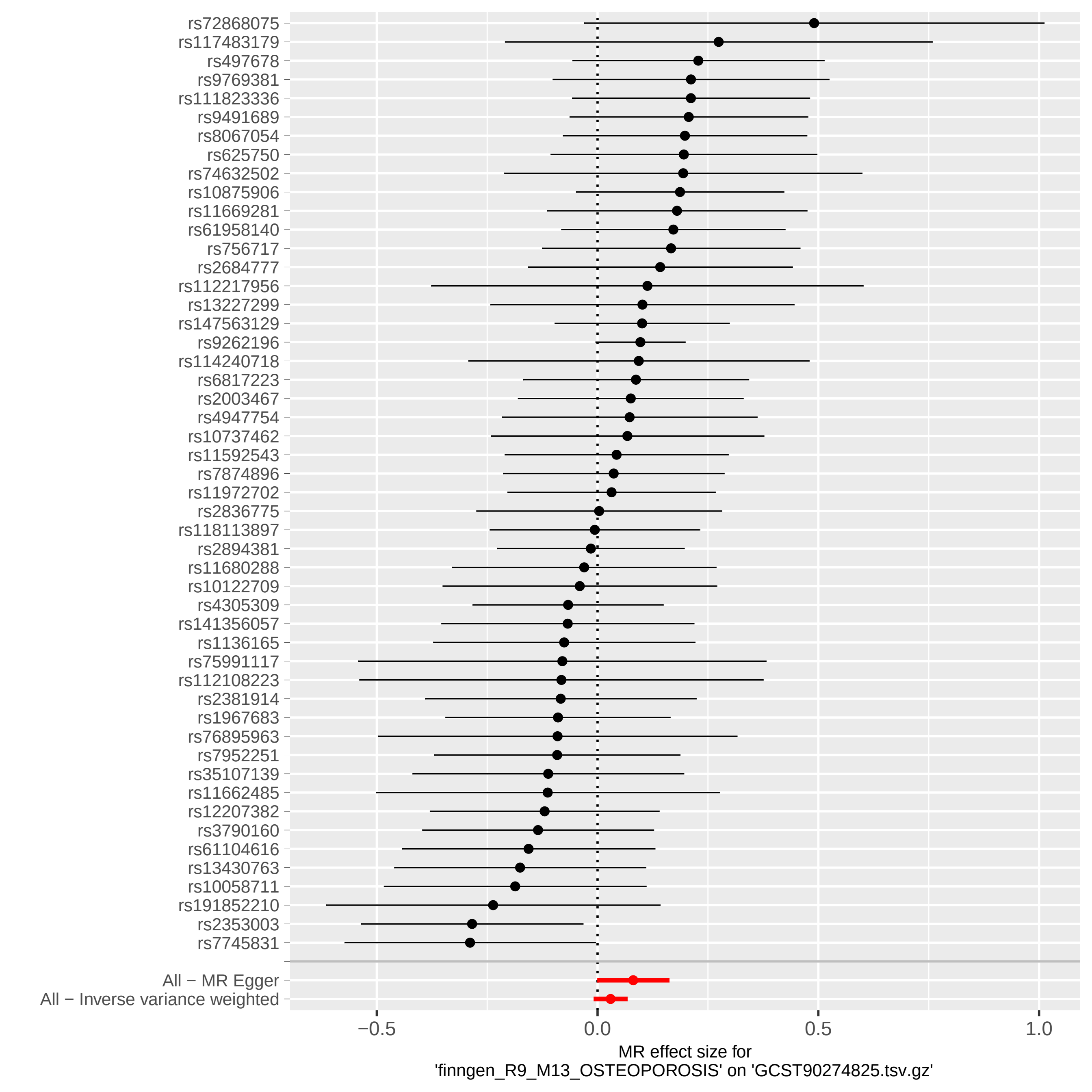
rs72868075
rs117483179
rs497678
rs9769381
rs111823336
rs9491689
rs8067054
rs625750
rs74632502
rs10875906
rs11669281
rs61958140
rs756717
rs2684777
rs112217956
rs13227299
rs147563129
rs9262196
rs114240718
rs6817223
rs2003467
rs4947754
rs10737462
rs11592543
rs7874896
rs11972702
rs2836775
rs118113897
rs2894381
rs11680288
rs10122709
rs4305309
rs141356057
rs1136165
rs75991117
rs112108223
rs2381914
rs1967683
rs76895963
rs7952251
rs35107139
rs11662485
rs12207382
rs3790160
rs61104616
rs13430763
rs10058711
rs191852210
rs2353003
rs7745831
All − MR Egger
All − Inverse variance weighted
−0.5
0.0
0.5
1.0
MR effect size for
'finngen_R9_M13_OSTEOPOROSIS' on 'GCST90274825.tsv.gz'
